# Caranan Fiber from *Mauritiella armata* Palm Tree as Novel Reinforcement for Epoxy Composites

**DOI:** 10.3390/polym12092037

**Published:** 2020-09-08

**Authors:** Andressa Teixeira Souza, Raí Felipe Pereira Junio, Lucas de Mendonça Neuba, Verônica Scarpini Candido, Alisson Clay Rios da Silva, Afonso Rangel Garcez de Azevedo, Sergio Neves Monteiro, Lucio Fabio Cassiano Nascimento

**Affiliations:** 1 Department of Materials Science, Military Institute of Engineering-IME, Rio de Janeiro 22290-270, Brazil; andressa.t.souza@gmail.com (A.T.S.); raivsjfelipe@hotmail.com (R.F.P.J.); lucasmneuba@gmail.com (L.d.M.N.); lucio@ime.eb.br (L.F.C.N.); 2 Materials Science and Engineering, Federal University of Para-UFPA, Rodovia BR-316, km 7.5-9.0, Centro, Ananindeua, 67000-000, Brazil; scarpini@ufpa.br (V.S.C.); alissonrios@ufpa.br (A.C.R.d.S.); 3 Department of Agricultural Engineering and Environment, Federal Fluminense University—UFF, Rua Passo da Pátria, 156, São Domingo, Niteroi, Rio de Janeiro 24210-240, Brazil; afonso.garcez91@gmail.com

**Keywords:** caranan fibers, natural fiber composite, tensile properties, thermal analysis

## Abstract

A growing environmental concern is increasing the search for new sustainable materials. In this scenario, natural lignocellulosic fibers (NLFs) became an important alternative to replace synthetic fibers commonly used as composites reinforcement. In this regard, unknown NLFs such as the caranan fiber (*Mauritiella armata*) found in South American rain forests revealed promising properties for engineering applications. Thus, for the first time, the present work conducted a technical characterization of caranan fiber-incorporated composites. Epoxy matrix composites with 10, 20 and 30 vol% of continuous and aligned caranan fibers were investigated by tensile tests, thermogravimetric analysis (TGA) and differential scanning calorimetry (DSC). Composites with more than 10% vol of caranan fibers significantly increase the elastic modulus and toughness in comparison to the neat epoxy. Indeed, the composite with 30 vol% was 50% stiffer, 130% tougher, and 100% stronger, which characterized an effective reinforcement. As for the elastic modulus, total strain and tensile toughness, there is a clear tendency of improvement with the amount of caranan fiber. The TGA disclosed the highest onset temperature of degradation (298 °C) with the least mass loss (36.8%) for the 30 vol% caranan fiber composite. It also displayed a higher degradation peak at 334 °C among the studied composites. The lowest glass transition temperature of 63 °C was obtained by DSC, while the highest of 113 °C by dynamic mechanical analysis (DMA) for the 30 vol% caranan composite. These basic technical findings emphasize the caranan fiber potential as reinforcement for polymer composites.

## 1. Introduction

Widely available in nature, natural lignocellulosic fibers (NLFs) are increasingly being considered sustainable alternatives for replacing synthetic fibers as polymer composite reinforcement in both scientific reviews [1,2,3,4,5,6,7,8,9,10,11] and possible industrial applications [12,13,14,15,16,17,18]. In fact, the specific properties (divided by the density) of the NLF composites are in some cases better than those of glass fiber composites (fiberglass) [19,20]. Moreover, Joshi et al. [21] propose that NLF composites are likely to be environmentally superior to fiberglass in most applications. It is also noteworthy that cellulose nanofibers extracted from NFL have recently been reported to substantially improve the mechanical properties and adhesion to a polymer nanocomposite [22]. In addition to superior specific properties, NLF composites have the advantage of fiber biodegradability, lower density, reduced process energy and cost effectiveness [23]. However, unattractive factors must be taken into account regarding the NLF behavior in terms of a large dispersion of physical properties, inhomogeneity inherent to the plant fiber structure and hydrophilicity [17,23]. Indeed, a relatively high level of moisture absorption might weaken the fiber adhesion to the hydrophobic polymer matrix [5,7]. Thermal stability is another issue of NLF processing, which is restricted by the fiber’s low temperature of cellulose degradation (~200 °C) as well as long periods of aging [24].

Today researchers are looking for new, less-known NLFs for developing improved polymer composites and their innovative application in engineering [25,26,27,28,29,30]. In this context, fibers extracted from the plant hard parts, like the stem or leaf-stalk (petiole), have better mechanical properties owing to greater cellulose content [31]. The caranan (English name adapted from the Portuguese *caranã*) fiber appears in the present scenario as a relatively unknown NLF extracted from leaf-stalk of a South American palm tree, *Mauritiella armata*. To our knowledge, the few scientific articles available on this palm tree are restricted to botanic characterization [32,33]. Except for possible use of its leaves for modest house roofing [34], no publication has yet been found in terms of caranan fiber application in composites. To explore this engineering potential, the present work investigates for the first time the basic mechanical and thermal properties of epoxy composites incorporated with up to 30 vol% of caranan fibers. The choice of the caranan fiber, in addition of its unknown potential for possible use in engineering composite, is due to its Amazonian origin. As a local rain forest product, which is collected without cutting the palm tree, the marketing of caranan fiber contributes to preserving the Amazon forest. The selection of epoxy as the composite matrix was based on its superior mechanical, thermal and corrosion resistance among most polymer resins as well as easy processing with NLFs [31,35]. Based on reviews in the literature [36,37], a possible application in multilayered armor systems (MAS) is another motivation to study this combination of less-known NLF polymer composites [38].

## 2. Materials and Methods

### 2.1. Materials

**Figure 1:**
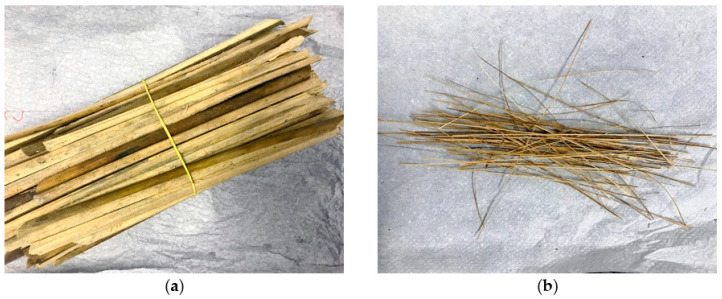
Defibrillation process of caranan fibers (**a**) leaf-stalk (**b**) defibrilated caranan fibers.

Leaf-stalks from *Mauritiella armata*, Figure 1a, used in this work to obtain caranan fibers, Figure 1b, were supplied by the Federal University of Para (UFPA, Belém Brazil). The polymer used to produce the composite matrix was an epoxy resin diglycidyl ether of the bisphenol A (DGEBA), produced by Dow Chemical, São Paulo, and distributed by Epoxyfiber, Rio de Janeiro, both in Brazil. The hardener applied to the resin was triethylene tetramine (TETA) with a stoichiometric ratio of 100 parts of epoxy to 13 parts of TETA. After manual extraction with a sharp razor, the caranan fibers, Figure 1a, were cleaned, defibrillated, cut to a length of 150 mm and dried in an oven at 70 °C for 24 h or until the weight of the fiber remains stable.

### 2.2. Composites Processing

**Figure 2:**
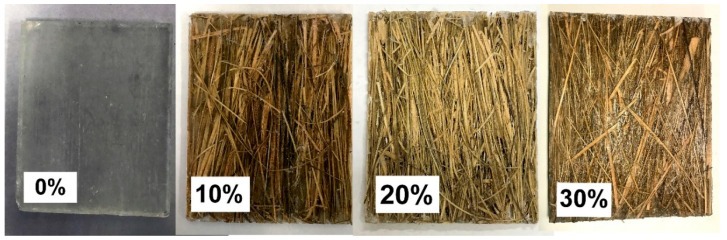
Composite plates of epoxy resin reinforced with caranan fiber.

For the preparation of composites, the proportions used were 10, 20 and 30% by volume of fiber, which correspond in absolute terms of mold loading to 14.12, 28.25 and 42.37 g, respectively. The density value of the epoxy resin was based on data reported elsewhere for the same epoxy [28]. For the DGEBA/TETA, an estimate of 1.11 g/cm^3^ was considered. The composite plates, Figure 2, were manufactured in a steel mold with an internal volume of 180 cm^3^ and dimensions of 15 × 12 × 1 cm^3^. With the aid of a SKAY hydraulic press (Skay Industry, São Paulo, Brazil) and a load of 5 tons for 24 h, the final plate was obtained.

### 2.3. Density and Porosity Measurements

Density and porosity of the neat epoxy and different composites were obtained by precise measurement of volume by the Archimedes methods and geometric dimensions using a caliper with 0.01 mm of precision. Weight measurements was performed in a 0.001 g of precision analog scale.

### 2.4. Tensile Tests

**Figure 3:**
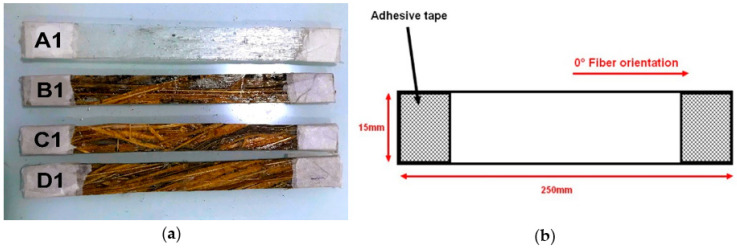
Specimen for tensile test of caranan fiber epoxy composites (**a**) 0% specimens, A1; 10%, B1; 20%, C1 and 30%, D1 (**b**) dimensions according to the standard (ASTM D3039) [39].

Tensile tests were performed according to the ASTM D3039 standard [39], in an Instron universal equipment (Instron, Norwood, MA, USA) model 3365, with a 25 kN load cell. The test crosshead speed was 2 mm/min. Four different composite plates were produced: 10, 20 and 30 vol% of fibers plus a neat epoxy (0% fiber) plate for control. All plates were cut to the dimensions indicated by the standard [39], shown in Figure 3. Seven specimens were tested for each composition.

### 2.5. Thermal Analysis

To analyze the thermal stability of the composites, thermogravimetric analyses (TGA) and differential scanning calorimetry (DSC) were performed according to the ASTM E1131 standard [40]. The equipment used was a Shimadzu, model DTG-60H (Shimadzu Precision Instruments Inc., Long Beach, CA, USA). Samples with approximately 10 mg were reduced to particles and placed in a platinum crucible. The atmosphere used was nitrogen with a flow rate of 50 mL/min, heating rate 10 °C/min, and temperature range from 10 to 600 °C for the TGA and from 25 to 250 °C for DSC.

The following variables were obtained during the TGA test: loss of mass, temperature at the beginning of abrupt loss of mass (T_onset_), and temperature at maximum rate of mass loss, associated with the derivative thermogravimetric (DTG) peak, as well as the characteristic thermal events. In addition, it was possible to disclose a thermal stability relationship between the volume fractions of 10, 20 and 30 vol% and that for the neat epoxy.

### 2.6. Micrography Analysis

Caranan fiber morphology was analyzed by scanning electron microscopy (SEM) in order to verify details such as surface, cross section, lumen and microfibrils. For this, the fiber was cryogenically fractured to preserve the relative shape and dimensions. The samples were fixed on a carbon ribbon and later sputtered with gold. The equipment used for metallization was a vacuum desk V by Denton, TX, USA, while for the images, a Quanta FEG 250 model FEI microscope (Field electron and Ion Co., Hillsboro, OR, USA). The working distance used was 10 mm and secondary electrons accelerated with 15 kV.

### 2.7. Dynamic Mechanical Analysis

Dynamic mechanical analysis (DMA) was carried out in order to identify the viscoelastic behavior of the material, as well as important parameters such as the glass transition temperature of the composites. The samples used in the percentages of 0, 10, 20 and 30 vol% followed the ASTM D4065 standard [41] and the test mode was two points bending for samples fixed by one end. The equipment used was a model DMA Q800, from TA Instruments (New Castle, DE, USA) and the samples dimensions 46 × 12 × 3 mm, from which curves of storage modulus, loss modulus and tangent delta were recorded.

## 3. Results and Discussions

### 3.1. Materials Basic Characterization

Table 1 presents calculated density for the neat epoxy and different caranan fiber epoxy composites. In this table is also presented the corresponding porosity for each of these investigated materials.

**Figure 4:**
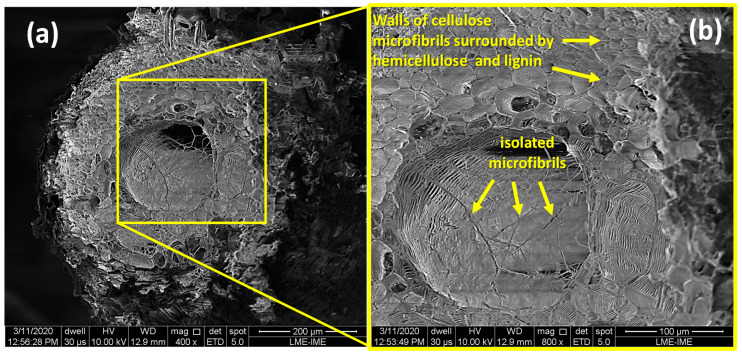
Scanning electron microscope (SEM) images: (**a**) 400× and (**b**) 800× of the cross section of a caranan fiber.

SEM images of the caranan fiber cross section are shown in Figure 4. It can be seen that it presents structures similar to those of other NFLs [23,24], with a lumen in the center, surrounded by several walls formed by microfibrils of semi-crystalline cellulose incorporated into a matrix of hemicellulose and lignin. The structure has an almost circular shape. In addition, its low density of 0.66 g/cm^3^, in comparison with other fibers [27,41,42,43,44,45], could be partially explained due to the large amount of lumen and the thin hollow structure of the cell wall.

A preliminary characterization of the caranan fiber/epoxy bonding performed by pullout test provided a relatively high interfacial shear strength of 17 MPa. Therefore, one should expect a good fiber/matrix adhesion and a caranan fiber reinforcement potential.

### 3.2. Tensile Test

**Figure 5:**
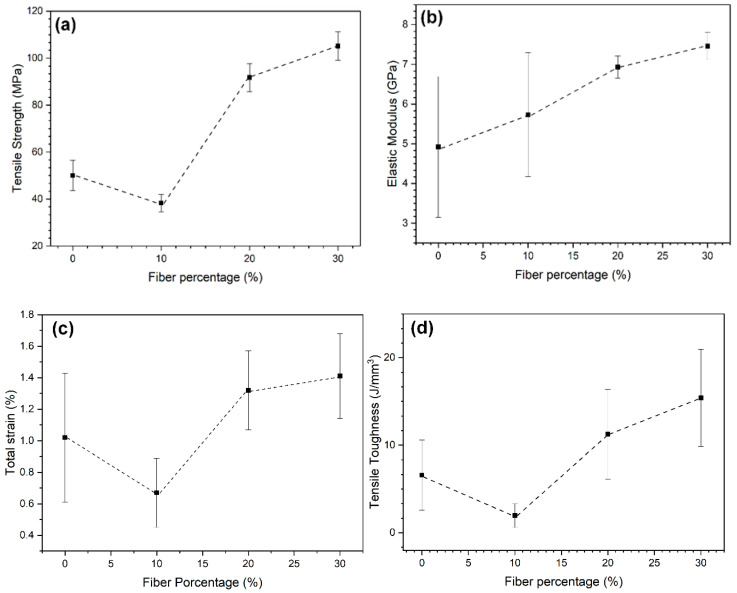
Variation of (**a**) tensile strength (**b**) elastic modulus (**c**) total elongation (**d**) tensile toughness for neat epoxy (0%) and caranan fiber composites.

Table 2 presents the basic tensile properties of neat epoxy (0%) and caranan fiber epoxy composites. Based on the results in Table 2, Figure 5 shows the variation in the tensile properties of the composites with the volume fraction of caranan fiber.

For the 20 and 30 vol% caranan fiber composites, it was found that the mechanical strength increases with the percentage of fibers, confirming the hypothesis that the fiber works as an effective reinforcement. As already reported in the literature [46], this is a consequence of the mechanisms of rupture of the fibers and their debonding at the fiber–matrix interface. However, with the addition of only 10 vol%, the fiber acted as a filler or defect, reducing the tensile strength of the epoxy matrix. On the other hand, there is an increase in the modulus of elasticity as well in the total elongation and tensile toughness (absorbed energy) for 30 vol% caranan fiber composite as compared to the neat (0%) epoxy.

As shown in Figure 5 the incorporation of higher amounts of caranan fibers improves the tensile properties. Indeed, as presented in Table 2, the incorporation of 30 vol% of caranan fibers tends to increase not only the strength (100%), stiffness (50%) and toughness (130%) of the epoxy matrix but also its total elongation by 40%. This proves the reinforcement behavior of the fibers, which also contributes to the ductility of the epoxy matrix. Regarding the results of tensile strength in Table 2 and Figure 5a for the 30% vol% caranan fiber, it is relevant to establish a comparison with 30 vol% glass fiber reinforcement epoxy composite, as was previously reported for a similar curaua epoxy composite [19]. The tensile strength of 131 MPa for the glass fiber composite when divided by the composite density (1.55 g/cm^3^) gives a specific strength (85 MPa) lower than that of the caranan fiber composite (105/0.975 = 108 MPa) Therefore, for this volume fraction, the caranan composite might be a viable substitute for fiberglass.

**Figure 6:**
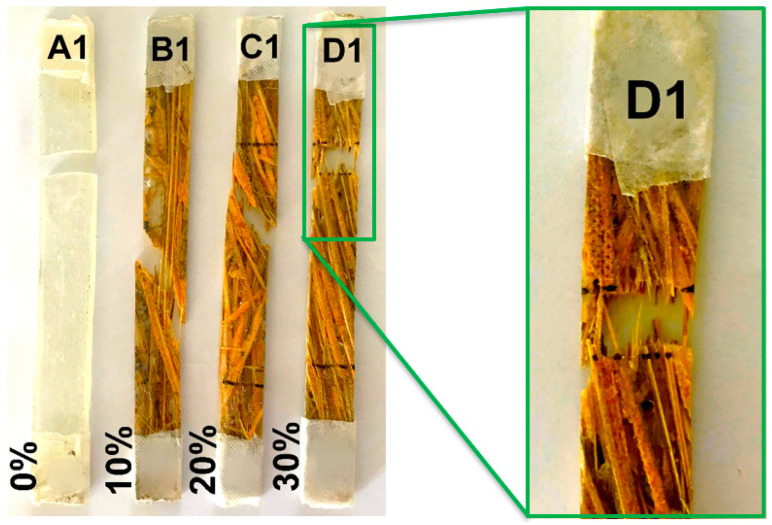
Typical broken specimens of the caranan fiber epoxy composite after the tensile test.

Figure 6 shows typical broken specimens of each composition ruptured in tensile tests.

**Figure 7:**
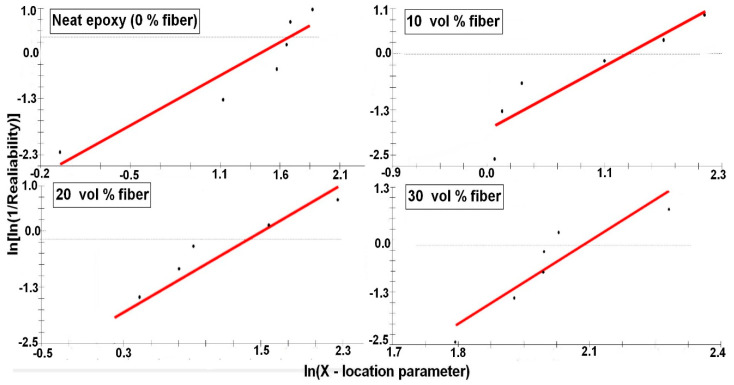
Tensile strength Weibull plots for the epoxy composites reinforced with different volume fractions of caranan fiber.

**Figure 8:**
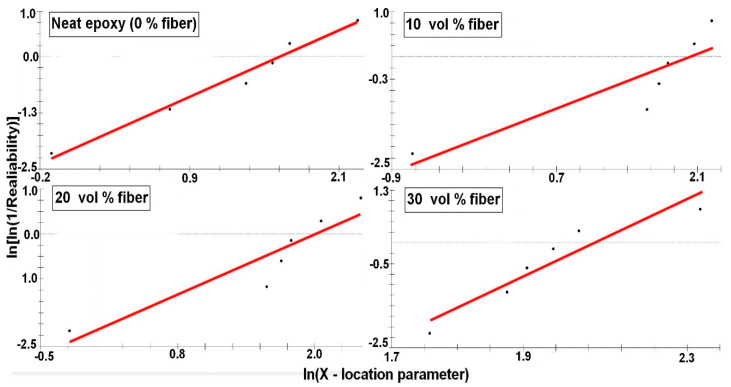
Elastic modulus Weibull plots for the epoxy composites reinforced with different volume fractions of caranan fiber.

**Figure 9:**
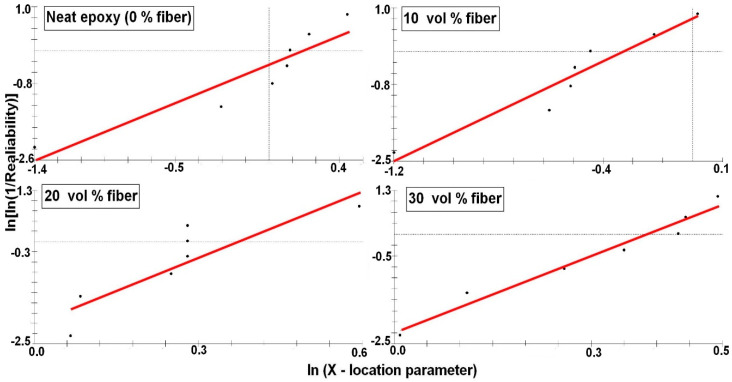
Total elongation Weibull plots for the epoxy composites reinforced with different volume fractions of caranan fiber.

**Figure 10:**
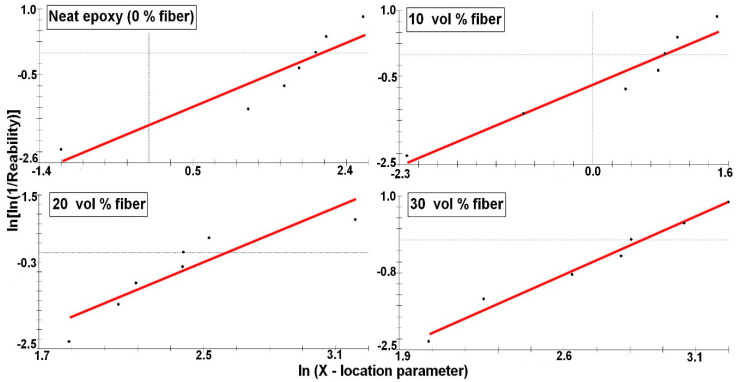
Tensile toughness Weibull plots for the epoxy composites reinforced with different volume fractions of caranan fiber.

To confirm the reinforcement effect provided by the caranan fibers, the properties obtained in tensile tests of each composite, Figure 5, were statistically analyzed by the Weibull method. Figure 7, Figure 8, Figure 9 and Figure 10 shows the statistical reliability versus the corresponding Weibull location parameter. Straight lines adjust the corresponding points for the same property in the different composites.

Table 3 presents the values of the Weibull parameters (β and R^2^), as well as their characteristic stress θ. The R^2^ values indicate that all the results obtained are statistically reliable. The observed θ values are similar to those found for corresponding properties in Table 2. Here, it is important to note that the relatively low value of the Weibull modulus β is due to the heterogeneous characteristics related to the biological process of formation of any NLF [23,47], such as caranan in the present work.

### 3.3. Thermogravimetric Analysis

**Figure 11:**
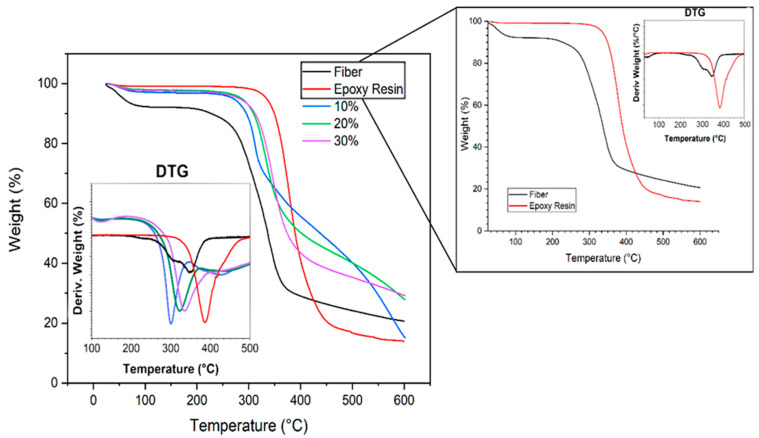
Thermogravimetric analysis (TGA) and derivative thermogravimetric (DTG) curves of caranan fiber, epoxy resin and their composites.

The results of TG/DTG curves evaluating the influence of the volume fraction of caranan fibers on the thermal stability of the epoxy composites are shown in Figure 11.

As already described in the literature [48,49] the greater the volume of NLFs in the composite, the higher its temperature of onset degradation (T_onset_). Table 4 indicates that the thermal stability of the composites is directly related to the amount of fibers added as reinforcement. In the curves of Figure 11 this become evident by the DTG peaks of the composites, which are moving to the right and increasing their thermal stability. The initial small weight loss (<5%) is associated with water release below 150 °C, which is a common feature of composites reinforced with natural fibers [48,49].

With the increase in caranan fiber content, the maximum degradation temperature rises and the weight loss % significantly decreases. It is worth noting that the onset of degradation occurs between 200–300 °C, and is attributed to the decomposition of cellulose and hemicellulose. As for the lignin, its thermal decomposition occurs in a broader range that initiates earlier but extends to higher temperature [48]. According to the TG/DTG curves in the inset in Figure 11, the complete thermal degradation of caranan fibers and epoxy matrix are expected to occur above 400 and 500 °C, respectively. The weight loss around 80% at 600 °C in Figure 11 corresponds to formation of a relatively higher amount of char. At even higher temperatures, one would expect a final char residue of around 10% for all investigated materials [50]. As such, one may consider 200 °C as the maximum working temperature for the caranan fiber epoxy composite.

### 3.4. Differential Scanning Calorimetry Analysis

**Figure 12:**
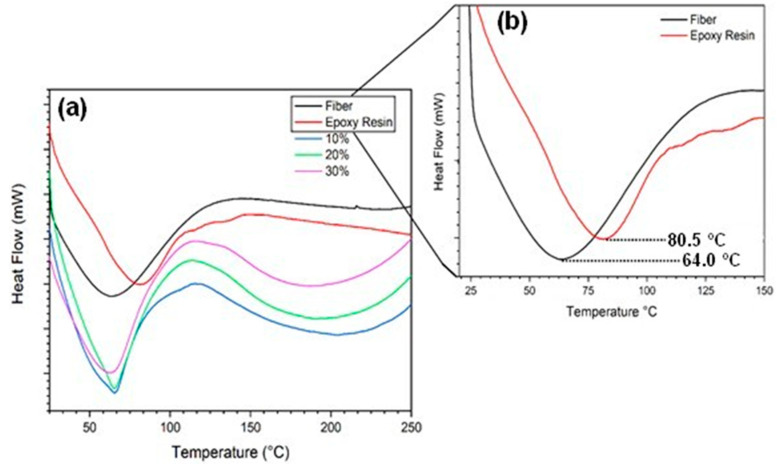
Differential scanning calorimetry (DSC) curves of (**a**) caranan fiber, epoxy resin and their composites (**b**) magnified endothermic peaks for the caranan fiber and neat epoxy.

The DSC analysis, Figure 12, is an important technique used and disseminated for the characterization and identification of polymers [49]. From the DSC curves, it was possible to determine the glass transition temperature (T_g_) of the composites studied in this work.

Table 5 shows that the T_g_ of the epoxy is around 81 °C while that of the caranan fiber would be at 64 °C. However, the endothermic event might also be related to the fiber moisture release. It is known that the T_g_ of a polymer depends on the mobility of the chain segment of the macromolecules in the polymeric matrix [49,50,51,52]. In this case, the reinforcing effect of the caranan fibers interfere with the movement of the polymeric chains and, thus, decreases the T_g_ of the composite. Within the investigated temperature range, the curves did not present any relevant event for this analysis, which suggests that the glass transition temperature of the composite shifted because of the presence of the reinforcement. Exothermic peaks at around 114 °C are related to a possible curing of the polymer matrix [28].

### 3.5. Dynamic Mechanical Analysis

**Figure 13:**
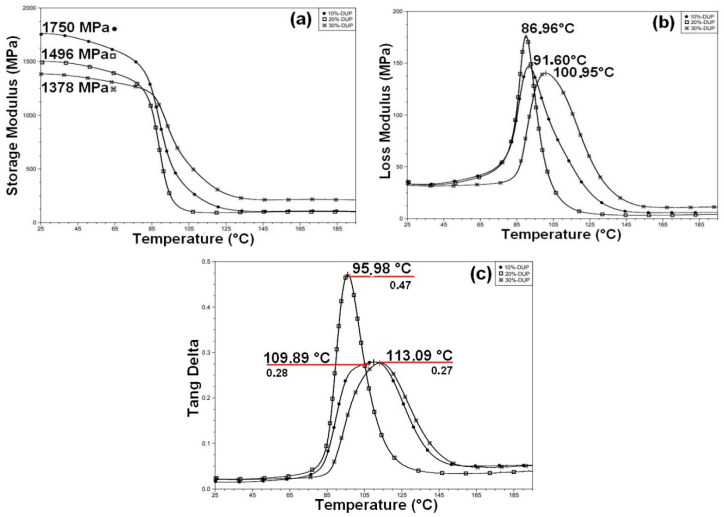
Dynamic mechanical analysis (DMA): (**a**) storage modulus; (**b**) loss modulus and (**c**) tangent delta for the caranan fiber composites.

Figure 13 shows the curves of storage modulus, G’, loss modulus, G’’, and tangent delta, tan δ, of the neat epoxy [53] and different caranan fiber composites, from 25 up to 200 °C.

The G’ results in Figure 13a reveal an improvement of the storage modulus with the incorporation of caranan fiber, which confirms the reinforcement effect owing to its better interaction with polymer matrix [30]. As for the G’’ results in Figure 13, the incorporation of caranan fiber displaces the peak of loss modulus to higher temperatures as compared to that of the neat epoxy reported elsewhere [53]. According to Mohanty et al. [54], the G’’ peak is related to the structural relaxation and might be assigned to T_g_. A similar situation occurs for the Tan δ peaks in Figure 13c. Table 6 presents the main DMA parameters associated with the composite curves in Figure 13 and neat epoxy [53]. A comparison between the values of T_g_ obtained from DSC in Table 5 with those in Table 6, indicates that the DMA Tan δ values correspond to the upper limits of T_g_ [54].

## 4. Conclusions

The first technological results on caranan fibers extracted from the leaf-stalk of *Mauritiella armata* and their 10, 20 and 30 vol% epoxy composites are revealed. A caranan fiber density of 0.66 g/cm^3^ was found as one of the lowest for natural lignocellulosic fiber (NLFs), which contribute to lower densities: 1.065, 1.020 and 0.975 g/cm^3^ of the 10, 20 and 30 vol% composites, respectively. Porosities of composites (9.32–11.90%) increase in comparison with that of neat epoxy (9.01%) due to the porous microstructure of the caranan fiber. The SEM micrograph of the caranan fiber showed a morphological porous aspect similar to that already reported for other NLFs with a cross section tending to a circular shape. In addition, cylindrical elements close to the lumen are attributed to the cellulose-based microfibrils, as well hemicellulose and lignin, that make up the fiber walls. Moreover, 30 vol% caranan fiber significantly improves (100%) the tensile strength of epoxy resin composites characterizing a reinforcement effect. Modulus of elasticity (50%), total elongation (40%) and tensile toughness (130%) of the composite also increase with 30 vol% of caranan fibers incorporated into the matrix. The thermal analysis results show that the maximum working temperature of the fiber and the composite is 200 °C. Furthermore, the thermal stability of composites is directly related to the amount of fibers added as reinforcement. The DSC and DMA tests made it possible to determine the limits of the glass transition temperature (T_g_) of epoxy composites reinforced with caranan fiber. The interval of 62–65 °C was found to be lower limits, and 96–113 °C to be upper limits, for T_g_. For our particular research, such properties regarding the caranan composites enable potential applications in multilayered armor systems against high-energy projectiles. However, the lack of information in the literature about the fiber strength and its epoxy composite’s ballistic performance makes it necessary to conduct a more comprehensive study by means of impact tests and residual velocity.

## Figures and Tables

**Table 1:** Results for density and porosity test of the epoxy-caranan composite.

Fiber Volume (%)	Density (g/cm^3^)	Porosity (%)
0	1.110	9.01
10	1.065	9.32
20	1.020	9.74
30	0.975	11.90

**Table 2:** Results for tensile test of the epoxy–caranan composite.

Fiber Volume (%)	Tensile Strength (MPa)	Elastic Modulus (GPa)	Total Elongation (%)	Tensile Toughness (J/mm^3^)
0	50.0 ± 6.4	4.9 ± 1.7	1.0 ± 1.0	6.6 ± 4.0
10	38.3 ± 3.6	5.7 ± 1.5	0.7 ± 0.6	1.7 ± 1.35
20	91.7 ± 5.9	6.9 ± 0.2	1.3 ± 1.3	11.3 ± 5.14
30	105.2 ± 6.0	7.4 ± 0.3	1.4 ± 1.4	15.4 ± 5.54

**Table 3:** Weibull parameters for the measured properties by the tensile strength test of epoxy composites reinforced with caranan fiber Weibull analysis.

Fiber Volume (%)	Tensile Strength			Modulus of Elasticity			Total Strain			Tensile Toughness		
	R^2^	β	ϴ	R^2^	β	θ	R^2^	β	θ	R^2^	β	θ
0	0.88	1.66	56.67	0.86	1.69	1.22	0.94	0.97	8.51	0.86	0.82	8.38
10	0.85	3.28	42.95	0.92	2.76	0.76	0.92	3.72	7.02	0.94	0.81	2.32
20	0.87	5.02	99.28	0.79	5.29	1.46	0.90	3.22	8.06	0.82	2.35	12.86
30	0.84	5.59	113.5	0.97	5.27	1.53	0.89	5.23	8.43	0.98	2.54	17.53

**Table 4:** Information about TGA analysis in different conditions.

Condition	T_onset_ (°C)	Weight Loss (%)	Maximum Degradation Temperature (°C)
Caranan Fiber	257.5	69.7	339.0
Epoxy Resin	286.5	77.6	386.3
10%	210.5	49.6	309.4
20%	272.6	42.6	323.5
30%	298.1	36.5	334.2

**Table 5:** Glass transition temperature (T_g_) for different material conditions.

Condition	T_g_ (°C)
Caranan Fiber	64
Epoxy Resin	81
10%	65
20%	65
30%	63

**Table 6:** DMA parameters.

Material	G’ (35 °C)	G’’ T_g_(°C)	Tan δ T_g_ (°C)	Reference
0% (epoxy)	1352	64	72	[53]
10%	1750	92	110	PW
20%	1496	87	96	PW
30%	1378	101	113	PW

PW = Present Work.

## References

[1] Hassan K.M., Horvath P.G., Alpár T. (2020). Potential Natural Fiber Polymeric Nanobiocomposites: A Review. Polymers.

[2] Zhang Z., Cai S., Li Y., Wang Z., Long Y., Yu T., Shen Y. (2020). High performances of plant fiber reinforced Composites—A new insight from hierarchical microstructures. Compos. Sci. Technol..

[3] Sanjay M.R., Madhu P., Jawaid M., Senthamaraikannan P., Senthil S., Pradeep S. (2018). Characterization and properties of natural fiber polymer composites: A comprehensive review. J. Clean. Prod..

[4] Pickering K.L., Efendy M.A., Le T.M. (2016). A review of recent developments in natural fibre composites and their mechanical performance. Compos. Part A Appl. Sci. Manuf..

[5] Güven O., Monteiro S.N., Moura E.A., Drelich J.W. (2016). Re-emerging field of lignocellulosic fiber–polymer composites and ionizing radiation technology in their formulation. Polym. Rev..

[6] Mohammed L., Ansari M.N.M., Pua G., Jawaid M., Islam M.S. (2015). A Review on Natural Fiber Reinforced Polymer Composite and Its Applications. Int. J. Polym. Sci..

[7] Faruk O., Bledzki A.K., Fink H.P., Sain M. (2014). Progress report on natural fiber reinforced composites. Macromol. Mater. Eng..

[8] Thakur V.K., Thakur M.K., Gupta R.K. (2014). Raw natural fiber–based polymer composites. Int. J. Polym. Anal. Charact..

[9] Shah D.U. (2013). Developing plant fibre composites for structural applications by optimising composite parameters: A critical review. J. Mater. Sci..

[10] Faruk O., Bledzki A.K., Fink H.-P., Sain M. (2012). Biocomposites reinforced with natural fibers: 2000–2010. Prog. Polym. Sci..

[11] Zini E., Scandola M. (2011). Green composites—An overview. Polym. Compos..

[12] Potluri R., Krishna N.C. (2020). Potential and Applications of Green Composites in Industrial Space. Mater. Today Proc..

[13] Youssef A.M., El-Sayed S.M. (2018). Bionanocomposites materials for food packaging applications: Concepts and future outlook. Carbohyd. Polym..

[14] Di Bella G., Fiore V., Galtieri G., Borsellino C., Valenza A. (2014). Effects of natural fibres reinforcement in lime plasters (kenaf and sisal vs. Polypropylene). Constr. Build. Mater..

[15] Majeed K., Jawaid M., Hassan A., Abu Bakar A., Abdul Khalil H.P.S., Salema A.A., Inuwa I. (2013). Potential materials for food packaging from nanoclay/natural fibres filled hybrid composites. Mater. Des..

[16] Dittenber D.B., GangaRao H.V.S. (2012). Critical review of recent publications on use of natural composites in infrastructure. Compos. Part A Appl. Sci. Manuf..

[17] Thomas N., Paul S.A., Pothan L.A., Deepa B., Kalia S., Kaith B.S., Kaur I. (2011). Natural fibers: Structure, properties and applications. Cellulose Fibers: Bio-and Nano-Polymer Composites.

[18] Holbery J., Houston D. (2016). Natural-fiber-reinforced polymer composites in automotive applications. JOM-US.

[19] Maciel N.D.O.R., Ferreira J.B., da Vieira J.S., Ribeiro C.G.D., Lopes F.P.D., Margem F.M., Silva L.C.d. (2018). Comparative tensile strength analysis between epoxy composites reinforced with curaua fiber and glass fiber. J. Mater. Res. Technol..

[20] Wambua P., Ivens J., Verpoest I. (2003). Natural fibres: Can they replace glass in fibre reinforced plastics?. Compos. Sci. Technol..

[21] Joshi S., Drzal L., Mohanty A., Arora S. (2004). Are natural fiber composites environmentally superior to glass fiber reinforced composites?. Compos. Part A Appl. Sci. Manuf..

[22] Tarrés Q., Oliver-Orteza H., Alcala M., Espinach F.X., Mutjé P., Delgado-Aguilar M. (2020). Research on the strengthening advantages on using cellulose nanofibers as polyvinyl alcohol reinforcement. Polymers.

[23] Monteiro S.N., Lopes F.P.D., Barbosa A.P., Bevitori A.B., Silva I.L.A.D., Costa L.L.D. (2011). Natural Lignocellulosic Fibers as Engineering Materials—An Overview. Metall. Mater. Trans. A.

[24] Hassan T., Jamshaid H., Mishra R., Khan M.Q., Petru M., Novak J., Choteborsky R., Hromasova M. (2020). Acoustic, Mechanical and Thermal Properties of Green Composites Reinforced with Natural Fibers Waste. Polymers.

[25] Da Costa Garcia Filho F., da Luz F.S., Oliveira M.S., Pereira A.C., Costa U.O., Monteiro S.N. (2020). Thermal behavior of graphene oxide-coated piassava fiber and their epoxy composites. J. Mater. Res. Technol..

[26] De Mendonça Neuba L., Pereira Junio R.F., Ribeiro M.P., Souza A.T., de Sousa Lima E., Garcia Filho F.D.C., Monteiro S.N., da Silva Figueiredo A.B.-H., de Oliveira Braga F., de Azevedo A.R.G. (2020). Promising mechanical, thermal, and ballistic properties of novel epoxy composites reinforced with cyperus malaccensis sedge fiber. Polymers.

[27] Reis R.H.M., Nunes L.F., Oliveira M.S., de Veiga V.F., Garcia Filho F.D.C., Pinheiro M.A., Monteiro S.N. (2020). Guaruman fiber: Another possible reinforcement in composites. J. Mater. Res. Technol..

[28] Nascimento L.F.C., da Luz F.S., Costa U.O., Braga F.O., Lima Júnior É.P., Monteiro S.N. (2019). Curing Kinetic Parameters of Epoxy Composite Reinforced with Mallow Fibers. Materials.

[29] Da Demosthenes L.C.C., Nascimento L.F.C., Monteiro S.N., Costa U.O., da Garcia Filho F.C., da Luz F.S., Oliveira M.S., Ramos F.J.H.T.V., Pereira A.C., Braga F.O. (2019). Thermal and structural characterization of buriti fibers and their relevance in fabric reinforced composites. J. Mater. Res. Technol..

[30] Wang H., Memon H., Hassan E.A.M., Elagib T.H.H., Hassan F.E.A.A., Yu M. (2019). Rheological and dynamic mechanical properties of abutilon natural staw and polyactic acid biocomposites. Int. J. Polym. Sci..

[31] Jeyapragash R., Srinivasan V., Sathiyamurthy S. (2020). Mechanical properties of natural fiber/particulate reinforced epoxy composites—A review of the literature. Mater. Today Proc..

[32] Funk V., Hollowell T., Berry P., Kelloff C., Alexander S.N., Hollowell T.H., Kellof C.L. (2007). Checklist of the plants of the Guayana Shield (Venezuela: Amazonas, Bolivar, Delta Amacuro; Guyana, Surinam, French Guiana).

[33] Henderson H., Berry P.E., Holst B.K. (1997). Araliaceae in: Steyermark JA. Glora of the Venezuelan Guayana: Araliaceae—Cactaceae.

[34] Smith N. (2015). Mauritiella Armata. Palms and People in the Amazon.

[35] Neves A.C.C., Rohen L.A., Mantovani D.P., Carvalho J.P.R.G., Vieira C.M.F., Lopes F.P.D., Monteiro S.N. (2019). Comparative mechanical properties between biocomposites of Epoxy and polyester matrices reinforced by hemp fiber. J. Mater. Res. Technol..

[36] Nayar S.Y., Sultan M.T.B.H., Shenuy S.T., Kini C.R., Samant R., Shah A.U.M., Amunthakkaannan P. (2020). Potential of natural fibers in composites for ballistic applications—A review. J. Nat. Fibers.

[37] Benzait Z., Trabzon L. (2018). A review of recent research on materials used in in polymer-matrix composites for body armor application. J. Comp. Mater..

[38] Nascimento L.F.C., Louro L.H.L., Monteiro S.N., Lima E.P., Luz F.S. (2017). Mallow fiber-reinforced epoxy composites in multilayered armor for personal ballistic protection. JOM-US.

[39] (2017). ASTM D3039/D3039M-17. Standard (Test Method for Tensile Properties of Polymer Matrix Composite Materials.

[40] (2020). ASTM E1131-20. Standard Test Method for Compositional Analysis by Thermogravimetry.

[41] (2012). ASTM D4065. Standard Practice for Plastics: Dynamic Mechanical Properties: Determination and Report of Procedures.

[42] Hamad S.F., Stehling N., Holland C., Foreman J.P., Rodenburg C. (2017). Low-Voltage SEM of Natural Plant Fibers: Microstructure Properties (Surface and Cross-Section) and their Link to the Tensile Properties. Procedia Eng..

[43] Da Luz F.S., Ramos F.J.H.T.V., Nascimento L.F.C., da Silva Figueiredo A.B.H., Monteiro S.N. (2018). Critical length and interfacial strength of PALF and coir fiber incorporated in epoxy resin matrix. J. Mater. Res. Technol..

[44] Monteiro S.N., Margem F.M., Margem J.I., de Souza Martins L.B., Oliveira C.G., Oliveira M.P. (2014). Infra-Red Spectroscopy Analysis of Malva Fibers. Mater. Sci. Forum.

[45] Monteiro S.N., Satyanarayana K.G., Ferreira A.S., Nascimento D.C.O., Lopes F.P.D., Silva I.L.A., Portela T.G. (2010). Selection of high strength natural fibers. Matéria.

[46] Glória G.O., Teles M.C.A., Lopes F.P.D., Vieira C.M.F., Margem F.M., de Almeida Gomes M., Monteiro S.N. (2017). Tensile strength of polyester composites reinforced with PALF. J. Mater. Res. Technol..

[47] Wang F., Shao J. (2014). Modified Weibull Distribution for Analyzing the Tensile Strength of Bamboo Fibers. Polymers.

[48] Monteiro S.N., Calado V., Rodriguez R.J.S., Margem F.M. (2012). Thermogravimetric behavior of natural fibers reinforced polymer composites—An overview. Mater. Sci. Eng..

[49] Monteiro S.N., Calado V., Rodriguez R.J., Margem F.M. (2012). Thermogravimetric Stability of Polymer Composites Reinforced with Less Common Lignocellulosic Fibers—An Overview. J. Mater. Res. Technol..

[50] Mézáros E., Jakob E., Várhegyl G. (2007). Pyrolysis-GC/MS and TG/MS study of mediated laccase biodelinification of Eucalyptus globulus kraft pulp. J. Anal. Appl. Pyrol..

[51] Oliveira M.S., Pereira A.C., Monteiro S.N., da Costa Garcia Filho F., da Cruz Demosthenes L.C., Ikhmayies S., Li J., Vieira C., Margem J., de Oliveira Braga F. (2020). Thermal Behavior of Epoxy Composites Reinforced with Fique Fabric by DSC. Green Materials Engineering.

[52] Revanth J.S., Madhav V.S., Sai Y.K., Krishna D.V., Srividya K., Sumanth C.H.M. (2020). TGA and DSC analysis of vinyl ester reinforced by Vetiveria zizanioides, jute and glass fiber. Mater. Today Proc..

[53] Silva I.L.A., Bevitare A.B., Oliveira C.G., Margem F.M., Monteiro S.N. (2014). Dynamical-Mechanical Behavior of Epoxy Composites Reinforced with Jutefiber. Characterization of Minerals, Metals and Materials.

[54] Mohanty A.K., Misra M., Hinrichsen G. (2000). Biofiber, biodegradable polymers and Biocomposites: An overview. Macromol. Mater. Eng..

